# Direct Transformation of Metallized Paper into Al-Si Nano-Rod and Al Nano-Particles Using Thermal Micronizing Technique

**DOI:** 10.3390/ma11101964

**Published:** 2018-10-12

**Authors:** Zahra Karbalaei Mirza Shahrbabaki, Farshid Pahlevani, Narjes Gorjizadeh, Rumana Hossain, Mohammad Bagher Ghasemian, Vaibhav Gaikwad, Veena Sahajwalla

**Affiliations:** 1 Centre for Sustainable Materials Research and Technology, School of Materials Science and Engineering, UNSW Sydney, Sydney 2052, Australia; zkarbalaei74@gmail.com (Z.K.M.S.); n.gorjizadeh@unsw.edu.au (N.G.); r.hossain@unsw.edu.au (R.H.); v.gaikwad@unsw.edu.au (V.G.); veena@unsw.edu.au (V.S.); 2 Exchange Student from Department of Chemical Engineering, Faculty of Engineering, University of Tehran, Tehran 1417466191, Iran; 3 School of Materials Science and Engineering, UNSW Sydney, Sydney 2052, Australia; m.ghasemian@unsw.edu.au

**Keywords:** thermal micronizing, metallized paper, nano-particles, Al alloys

## Abstract

The abundant application of metallized paper and the quick growth of their wastes lead to the removal of a huge amount of valuable resources from economic cycle. In this work, for the first-time, the thermal micronizing technique has been used to directly transform the metallized paper wastes to Al-Si nano-rod and Al nano-particles for use as the input in different manufacturing sectors such as additive manufacturing or composite fabrication. Structure of metallized paper has been investigated using FT-IR analysis and first-principle plane-wave calculation. Then, based on the structure of metallized paper, thermal micronizing technique has been modified to directly transform this waste into nano materials. Structure of nano-particles and nano-rods has been investigated using SEM, TEM, and XPS analysis. Results showed two main Al-Si nano-rod and Al nano-particle morphologies created as a result of the different surface tensions, which facilitate their separation by Eddy current separation technique. These quick transformation and facile separation together make this technique a unique process to deal with this complex waste and producing value-added products which can re-capture these high value materials from waste and make the reforming economically viable.

## 1. Introduction

Metallised papers are substrates coated with a glossy aluminium layer which offer decorative and protective properties in different applications [1]. Metallised paper largely found their way to industries owing to their exceptional properties including excellent label ability, fast ink drying, high-speed labelling, sufficient transfer and release time, permanence of printing, resistance to alcohol, odour, lightness, etc. [1,2]. It is widely used as a high-end label in beer bottles and is particularly effective in enhancing the premium qualities of a product being labelled. Besides, metallised papers can also be found in chocolate wrappers, food products packaging, tobacco boxes, cigarette boxes, and decoration [3]. By the demanding progress in all forms of flexible packaging, metallised papers have attracted much interest and significant investments were focused in packaging and labelling industries to take advantage of metallised paper benefits. In the market today, the majority of metallised papers are consumed for labelling, specifically in products identification and decoration of drinks bottles and cigarette wraps. Geographically, in contrast to North America and Europe, there is a growing interest in metallised paper in South America, while this trend has remained at a high level in India and Asia Pacific. By increasing the price of aluminium and social and economic expenses of dealing with waste there is a growing demand for dealing with these wastes [4]. 

According to the reports provided by global metallised paper markets, millions of tons of metallized paper are produced worldwide annually and the percentage of metallised papers used in different industries is estimated as: Label (64%), packaging (29%), gift-wraps (5%), and the other applications (2%) [5].

Two types of processes can be carried out to produce metalized paper: Lamination and vacuum metallization. In lamination system, the process involves gluing a paper with aluminium with a thickness of 9–12 microns, while in vacuum metallization, a super fine layer of high vacuum aluminium of 0.08–0.1 g/m^2^ is deposited on one side coated substrate [1]. Chemically, metallized papers are complex composites of paper with a very thin layer of Al and other components including plastic substrate, coating, varnish, and lacquer. Due to this complex structure, this material cannot be recycled in conventional paper recycling process and there is no available process in recycling this material. In recent years it has been used as a source of waste to energy in combination with other waste but in this process the valuable aluminium and silicon, which, in this material, will be lost. To deal with this fast-growing waste and in order to capture this valuable material and bring it back to market, a new approach is needed.

Nowadays, wastes of metallised paper are disposed into the landfill. After a while, they release methane (CH_4_), which is a hazardous greenhouse gas. The growth of greenhouse gases causes the increase of the earth temperature. Beside the environmental issues, a massive amount of resources and energy are consumed through producing metallised paper causing a huge waste if metallised papers are disposed after the first use. Therefore, by recycling metallised papers to starting components or deforming their waste to useful materials, a lot of energy and financial resources can be saved. 

The aim of this study is to alleviate waste landfills issue by introducing a quick novel thermal transformation process, which is called thermal micronizing [6,7]. In this process, the reducing gasses, which are generated during degradation of paper at high temperature, will be used to produce nano-particles of Al and Al-Si alloys. At the same time, because this process is an exothermic process, generated energy can be used as a source of energy for this process or other applications. During this thermal micronizing process, metallized papers will be transformed to distinguished Al-based products, which are selectively separable to generate high-value aluminium based nano-particles.

## 2. Materials and Methods

### 2.1. Experimental Procedure

At the first stage the structure of metallised paper has been investigated using FT-IR. The FT-IR spectrum was obtained using a Perkin Elmer Spectrum 100 instrument, PerkinElmer, Waltham, MA, USA, equipped with a universal ATR attachment and diamond cell. The spectrum is the result of 16 scans in the wavenumber range of 4000–650 cm^−1^. 

A horizontal tube furnace, LABEC, Sydney, Australia was used for thermal micronizing process. The prepared metallised paper sample was pushed in graphite crucible before moving in the horizontal furnace by carbon rod. To prevent thermal shock, samples were placed in the cold zone with 250 °C for 10 min before pushing in the hot spot for performing thermal micronizing at different times; 5 and 60 min at 1500 °C to finalise the reaction. As the sample was very small in quantity, 10 g, and has been pushed directly to the pre-heated furnace of 1500 °C, the sample will reach to this temperature very fast. Subsequently, to avoid further oxidation, the samples were pulled back to the cold zone, 250 °C, for 10 min. To eliminate any remaining oxygen, pure argon gas with 1 l/min flow rate was used, which acts as carrier gas as well. The output gas during this heat treatment has been measured using an infrared gas analyser. 

An X-Ray diffraction instrument (XRD PANalytical Empyrean, Malvern Panalytical, Malvern, UK), unfiltered Co-Kα radiation, 45 kV and 40 mA) was used to identify the structural phases of products at the range of 2θ = 20°–120° with the step size of 0.02. The size and morphology of obtained materials were imaged by scanning electron microscopy (SEM AURIGA^®^), Carl Zeiss, Oberkochen, Germany. The weight percentage of elements in raw metallised papers were measured and compa, red by Laser-Ablation Inductively Coupled Plasma coupled to Mass Spectrometry (LAICPMS), PerkinElmer Waltham, MA, USA with NIST610 glass standard without an internal standard. 

### 2.2. Theoretical Method

The calculations were performed using first-principle plane-wave approach, based on the density-functional theory (DFT) [8]. We used the generalized gradient approximation (GGA) [9], with projector augmented-wave (PAW) pseudo-potentials as implemented in the Vienna ab initio simulation package (VASP) [10]. The cut-off kinetic energy for the plane-wave expansion was set to be 400 eV. Geometry of a Al(100) slab with 4 atomic layers was considered in a 3 × 3 supercell with periodic boundary conditions and a cellulose molecule was placed on top of it. A vacuum of 10 Å height in the supercell was used to prevent interactions in neighbouring supercells in the z direction. We calculated the equilibrium lattice parameter of FCC Al using a k-point mesh of 21 × 21 × 21 for the primitive cell of FCC Al, and a force convergence criterion of 0.001 eV/Å. The equilibrium lattice parameter of FCC Al was obtained to be 4.05 Å, in excellent agreement with experiment [11]. All the geometries were relaxed using a 1 × 1 × 1 k-point mesh and with the force criterion of 0.01 eV/Å. 

## 3. Results and Discussion

For quantitative analysis, a mixture of different types of metallized papers from local waste collection points were used as representative of actual metallised papers in landfills. Table 1 shows the average chemical composition of randomly selected mixture of metallized papers obtained by Laser-Ablation Inductively Coupled Plasma coupled to Mass Spectrometry (LAICPMS). 

**Figure 1:**
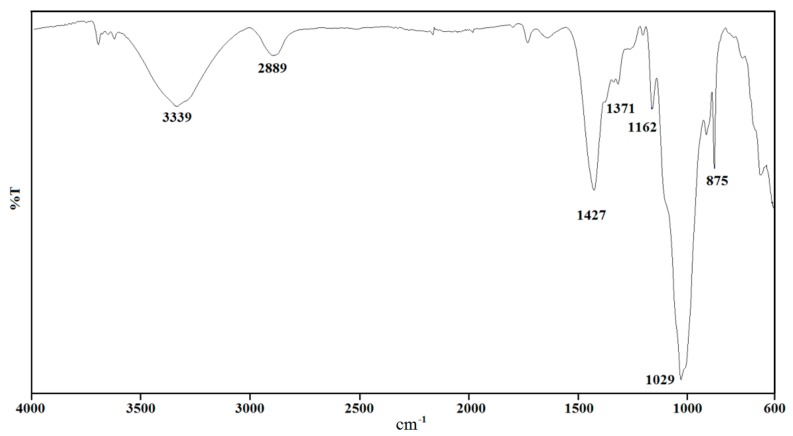
ATR FT-IR spectrum of untreated metal-coated paper.

FT-IR analysis was utilised to qualitatively identify the major components of the untreated metal-coated paper. The FT-IR spectrum of the same is presented in Figure 1. The peak centred at 1029 cm^−1^ can be attributed to the C-O stretching vibration due to the cellulose and hemicellulose (polysaccharide) molecules which usually the form the bulk of the paper matrix [12]. The C-O-C asymmetric stretching vibration from these molecules manifests itself in the region around 1160 cm^−1^ [12]. In addition, the peak at 2889 cm^−1^ can be attributed to a C-H stretching vibration, while the broad peak at 3339 cm^−1^ to an O-H stretching vibration in the cellulose/hemicellulose molecules [13].

As presented earlier by ICP analysis, the coated paper contains several elements including Ca and Si, the source of which is the clay, which is widely used during the paper manufacturing process. The peaks between 1371 and 1427 cm^−1^ can be attributed CO_3_ stretching vibration from the CaCO_3_ molecule. Similarly, the sharp peak at 875 cm^−1^ can be assigned to the CO_3_ bending vibration mode. The presence of CaO, which is the product of heat treated CaCO_3_, is also confirmed by the XRD analysis of the samples after heat treatment.

**Figure 2:**
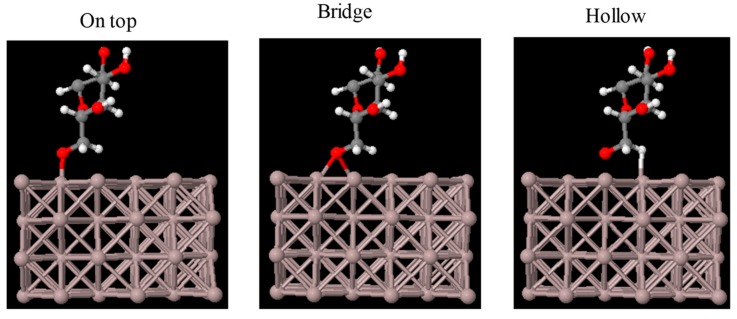
Relaxed Cellulose molecule deposited on an Al(100) surface at different deposition sites.

**Figure 3:**
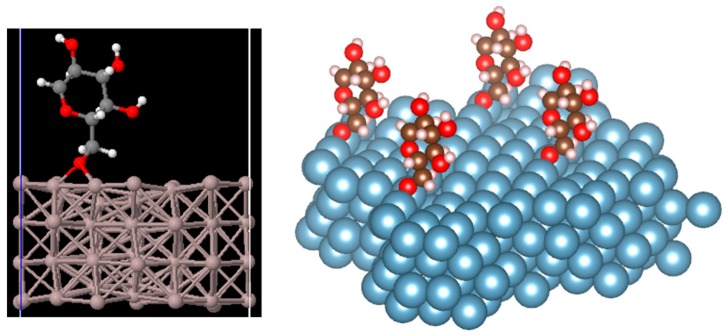
The optimized structure for the bridge site, which is a stable structure.

**Figure 4:**
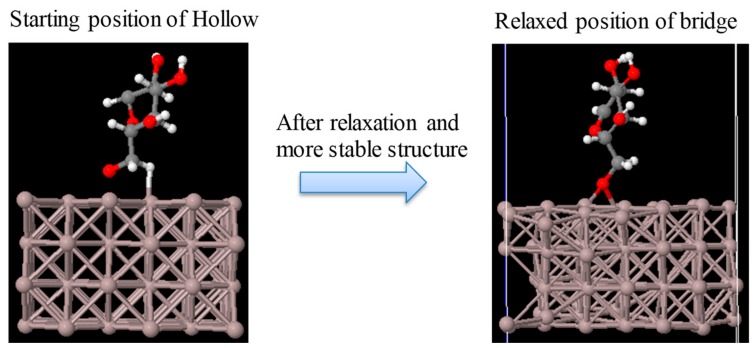
Moving of molecule from hollow position to bridge due to stability of this position.

The structure of a cellulose molecule deposited on an Al(100) surface was relaxed for three different deposition sites: Top, bridge, and hollow, as depicted in Figure 2. The structure of the molecule deposited on the bridge site was found to be the most favourable energetically. The optimized structure for the bridge site is shown in Figure 3. The binding energy between the molecule and the Al slab is calculated as $E_{b}=E_{Al+m}-\left(E_{Al}+E_{m}\right)$, where $E_{Al+m}$ is the total energy of the optimized system containing Al and the deposited molecule, $E_{Al}$ is the total energy of the Al slab, and $E_{m}$ is the total energy of the free molecule. All the three terms were calculated in a supercell with the same dimensions. The binding energy of cellulose was 3.96, 3.92, and 3.79 eV for the bridge, hollow, and top sites, respectively. This suggests that the bridge adsorption site is the most stable among these three. According to Figure 4, the starting position at the hollow site the molecule moved to the bridge site in the relaxed position with the O bonding to two Al atoms.

During the heat treatment of any material contain film or balk Al, its surface will be oxidized at heating temperatures less than 1000 °C [14]. Therefore, an annealing temperature of 1500 °C was chosen to guarantee a complete thermal transformation without oxidation of Al surface. 

**Figure 5:**
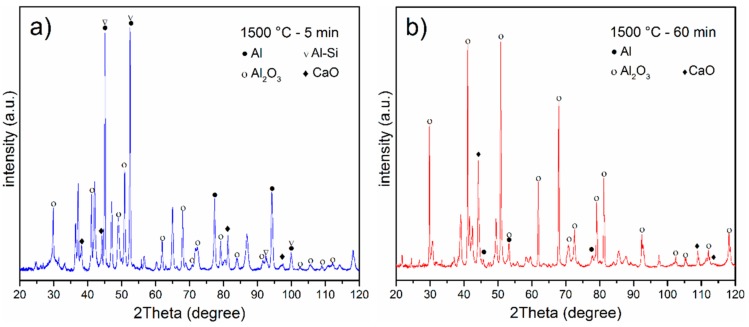
XRD patterns of annealed metallized paper at 1500 °C after (**a**) 5 and (**b**) 60 min.

Figure 5a,b shows the XRD results of the metallised paper annealed at 1500 °C for 5 min and 60 min, respectively. Different phases such as Al_2_O_3_ and Al-Si were produced at high temperature as evidenced by XRD analysis. The most important products in recycling of metallized papers are Al and Al-alloys, as this work focused on it. Recycled Al nano-particles has many application such as additive manufacturing or composite production [15]. Another application of aluminium powder is to be used in the paint manufacturing industry to create shiny colours [16]. On the other hand, Al-Si alloy is an appropriate alternative for producing high strength aluminium components through additive manufacturing [16]. Additionally, it improves wear resistant ability and the material fatigue limit for engine compounds [15].

In annealing process Al_2_O_3_ is produced in two ways. According to Equation (1), Al reacts with O_2_ to produce Al_2_O_3_, however, in the lack of oxygen aluminium provides required amount of oxygen by reacting with SiO_2_ as an alternative reaction, as shown in Equation (2) [17].

4Al + 3O_2_ → 2Al_2_O_3_, (1)

4Al + 3SiO_2_ → 3Si + 2Al_2_O_3_, (2)

**Figure 6:**
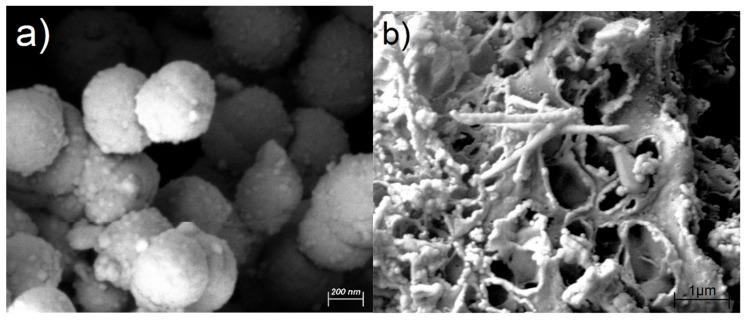
SEM images of (**a**) Al nono-particles and (**b**) Al-Si nano-rods produced from metallized paper annealed at 1500 °C for 5 min.

Morphology of Al-based products has been investigated using SEM analysis coupled with EDS. Figure 6 shows two different morphologies of the nano-particle of Al and nano-rod of Al-Si alloy formation. Due to different surface tension of Al and Al-Si alloy there is the possibility of formation of nano-particle shape, Figure 6a, and rod shape, Figure 6b, during heat treatment process. 

**Figure 7:**
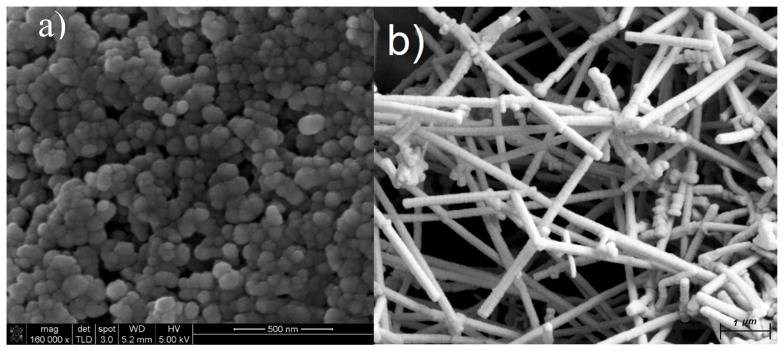
SEM images of (**a**) Al nano-particles and (**b**) Al-Si nano-rods produced from metallized paper annealed at 1500 °C for 60 min.

Figure 7, demonstrates the SEM results for annealed samples after 60 min at 1500 °C. Compared with Figure 6, the number of Al nano-particles and Al-Si nano-rod increased drastically because of more time of the formation and growth of nano-particles or nano-rods. The surface tension value for Al-Si alloys is higher than the surface tension of pure Al and Si at temperatures greater than 1300 °C [14]. The increase of annealing time causes the increase in diffusion of Si into the Al and formation of Al-Si nano-rods. 

**Figure 8:**
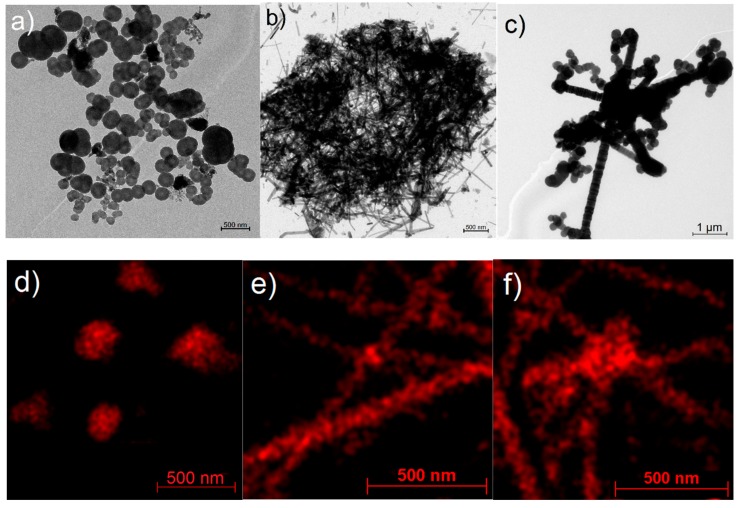
TEM images and Al EDS mappings of (**a**,**d**) round-shape, (**b**,**e**) rod-shape and (**c**,**f**) combined round/rod shape nanostructures.

Figure 8a,b shows the TEM images of round-shape and rod-shape nanostructures produced after 5 and 60 min of annealing at 1500 °C, respectively, while a combined structure of spheres and rods has been exhibited in Figure 8c. EDS mappings under TEM indicated that all of these nanostructures include Al uniformly which shows the formation of these alloy at high temperature and growth of nano-rods and nano-particles under thermal micronizing process. The combination of Al-alloys and carbon in an Al_2_O_3_ environment promotes the fully separation of tiny amounts of Al created through thermal transformation of metallized paper by Eddy technique.

**Figure 9:**
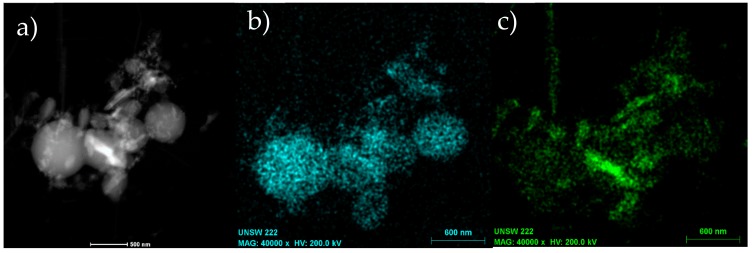
TEM backscattered images of a sample heat treated at 1500 °C for 5 min after separation of Al nanoparticles and Al-Si nano-rods. (**a**) TEM backscattered image, (**b**) EDS map of Al, and (**c**) EDS map of Si.

Figure 9 show the TEM and EDS analysis of a sample which contain Al nano-particles and Al-Si nano-rods at the same time which is for sample heat treated at 1500 °C for 5 min. This sample has been prepared after separation from other contamination and only contain Al nanoparticles and Al-Si nano-rods. EDS analysis clearly shows that nano-rods contain the Si which will be Al-Si alloy. 

**Figure 10:**
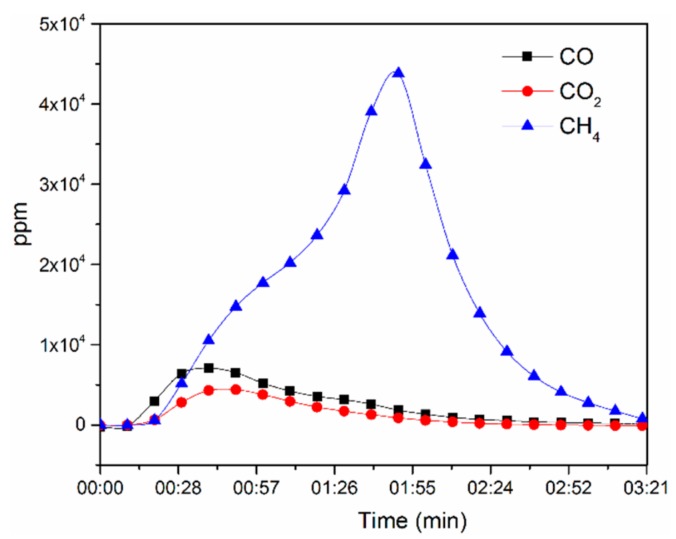
Gas analysis of metallized paper at 1500 °C.

The gas analysis was used to confirm the thermal micronizing process which is producing nano-particles due to formation of gas at high temperature and separating the liquid film of metal into nano-particles. Additionally, these reducing gasses will protect the Al nano-particles from oxidising. By having these nano-particles at higher temperature, Si starts to dissolve into the Al, and by formation of Al-Si alloy and due to different surface tension, it starts to form nano-rods. As graphed in Figure 10 demonstrate CH_4_, CO and CO_2_ were recognised as the main gases released in the range of 3 min after the combustion of the metallized paper in Ar atmosphere. Minor amounts of Co and CO_2_ gases were detected after ~30 s while a considerable amount of CH_4_ was observed after 1:55 min of thermal micronizing of metallized paper at 1500 °C. While the metallized paper was heat treated in an Ar atmosphere, the structural C-O, C-O-C and C-H bonds in the cellulose/hemicellulose molecules of the metallized paper, as ascertained by FT-IR, triggered the creation of CO, CO_2_, and CH_4_ gases. 

These results are indicating that using thermal micronizing process is the unique pass way to directly transform this waste into a valuable resource that can be used in other applications as a source of raw materials. In this process by fast heating of metallised paper to high temperature of 1500 °C, melting of Al film, degradation of paper layer, and formation of gasses will happen simultaneously, which results in the formation of Al nano-particles. Then, by continuing the reaction at high temperature Si from paper will selectively diffused into the liquid Al particles and form Al-Si alloy, which have different surface tensions and will form Al-Si nano-rods. If the reaction time increased the percentage of Al-Si nano-rods will increase as there is more time for this reaction. By controlling the reaction time, we can control the product to be Al nano-particles or Al-Si nano-rods. 

## 4. Conclusions

In this paper, for the first-time, thermal micronizing process has been used to directly transform the metallised paper wastes into Al nano-particles and Al-Si nano-rods. Effect of thermal transformation time on the formation mechanism and different structure of the produced Al-alloy was studied which indicated that at early state Al nano-particles will produce due to the thermal micronizing process and then through selective dissolution process Al-Si nano-rods will generated. The XRD results approved the presence of Al, Al_2_O_3_ and Al-Si in heat-treated metallised paper at 1500 °C. In addition, different structure of Al, Al nano-particles, and Al-Si nano-rods, contain homogenised Al in their structure, which is indicative of producing high quality and controlled structure of particles. These results are opening a new way for direct processing of these wastes, which contain high value materials. This new approach will change the economics of dealing with these wastes and open the pathway of using waste as input materials.

## Figures and Tables

**Table 1:** The results showing by LAICPMS of raw metallised paper.

	C *	Na	Al	Si	Ca	Ti
**Raw Metallised Paper**	31.8	0.405	6.50	16.82	13.70	0.10

* Carbon is indicative only.
